# Trends in working years lost due to different types of work disability and unemployment

**DOI:** 10.1093/eurpub/ckac130.203

**Published:** 2022-10-25

**Authors:** T Leinonen, E Viikari-Juntura, S Solovieva

**Affiliations:** Finnish Institute of Occupational Health, Helsinki, Finland

## Abstract

**Background:**

Little is known about working years lost (WYL) due to work disability and unemployment by industrial sector. This information would help in directing interventions promoting healthy working careers, knowing that the sectors have been differently affected by economic fluctuations and other changes in the labour market. We examined trends in WYL in the general Finnish population and by industrial sector in the period after the 2008 financial crisis with a particular focus on different types of work disability.

**Methods:**

Utilising register data on the Finnish working-age population and the Sullivan method, we calculated expected WYL due to sickness absence, other temporary work disability, partial disability retirement, full disability retirement, unemployment and other reasons in years 2010, 2013 and 2016 for the general male and female populations and by industrial sector.

**Results:**

In 2010, a 30-year-old person was expected to have around two-and-a-half to three WYL due to full disability retirement and unemployment until reaching age 65, depending on gender and the reason. By 2016, WYL due to full disability retirement decreased to less than two years and that due to unemployment increased to around four years among both genders. WYL due to sickness absence, other temporary work disability, partial disability retirement and other reasons remained relatively stable. The total WYL increased between 2010 and 2016 particularly among women. The differences by industrial sector in WYL were attributable more to unemployment than to the different work disability statuses.

**Conclusions:**

After the financial crisis unemployment appears to have replaced disability retirement as the most important reason for WYL. Furthermore, as individuals with a defined industrial sector are initially employed, sectoral differences in WYL are not largely attributable to disability retirement, disability pensioners typically having been outside the labour market for a long time.

**Key messages:**

- During economic downturns work disability may be increasingly disguised as unemployment, which should be considered when tackling problems of health and work ability of the labour force.
- Partly due to healthy worker effect, differences in working years lost by industrial sector are attributable to long-term work disability to a much smaller extent than to unemployment.

